# Pharmacokinetic Engineering of OX40-Blocking Anticalin Proteins Using Monomeric Plasma Half-Life Extension Domains

**DOI:** 10.3389/fphar.2021.759337

**Published:** 2021-10-25

**Authors:** Martin Siegemund, Prajakta Oak, Eva-Maria Hansbauer, Andrea Allersdorfer, Karoline Utschick, Alexandra Winter, Christina Grasmüller, Gunther Galler, Jan-Peter Mayer, Benjamin Weiche, Josef Prassler, Roland E. Kontermann, Christine Rothe

**Affiliations:** ^1^ Institute of Cell Biology and Immunology, University of Stuttgart, Stuttgart, Germany; ^2^ Pieris Pharmaceuticals GmbH, Hallbergmoos, Germany; ^3^ Stuttgart Research Center Systems Biology, University of Stuttgart, Stuttgart, Germany

**Keywords:** Anticalin protein, scaffold protein, half-life extension, albumin, IgG, binding domain, OX40, autoimmune disease

## Abstract

Anticalin^®^ proteins have been proven as versatile clinical stage biotherapeutics. Due to their small size (∼20 kDa), they harbor a short intrinsic plasma half-life which can be extended, e.g., by fusion with IgG or Fc. However, for antagonism of co-immunostimulatory Tumor Necrosis Factor Receptor Superfamily (TNFRSF) members in therapy of autoimmune and inflammatory diseases, a monovalent, pharmacokinetically optimized Anticalin protein format that avoids receptor clustering and therefore potential activation is favored. We investigated the suitability of an affinity-improved streptococcal Albumin-Binding Domain (ABD) and the engineered Fab-selective Immunoglobulin-Binding Domain (IgBD) SpGC3Fab for plasma Half-Life Extension (HLE) of an OX40-specific Anticalin and bispecific Duocalin proteins, neutralizing OX40 and a second co-immunostimulatory TNFRSF member. The higher affinity of ABD fusion proteins to human serum albumin (HSA) and Mouse Serum Albumin (MSA), with a 4 to 5-order of magnitude lower K_D_ compared with the binding affinity of IgBD fusions to human/mouse IgG, translated into longer terminal plasma half-lives (*t*_1/2_). Hence, the anti-OX40 Anticalin-ABD protein reached *t*_1/2_ values of ∼40 h in wild-type mice and 110 h in hSA/hFcRn double humanized mice, in contrast to ∼7 h observed for anti-OX40 Anticalin-IgBD in wild-type mice. The pharmacokinetics of an anti-OX40 Anticalin-Fc fusion protein was the longest in both models (*t*_1/2_ of 130 h and 146 h, respectively). Protein formats composed of two ABDs or IgBDs instead of one single HLE domain clearly showed longer presence in the circulation. Importantly, Anticalin-ABD and -IgBD fusions showed OX40 receptor binding and functional competition with OX40L-induced cellular reactivity in the presence of albumin or IgG, respectively. Our results suggest that fusion to ABD or IgBD can be a versatile platform to tune the plasma half-life of Anticalin proteins in response to therapeutic needs.

## Introduction

The receptor/ligand pairs of the Tumor Necrosis Factor Receptor Superfamily (TNFRSF) including OX40/OX40L not only have a prominent role as controllers of T lymphocyte activation and survival, but are also associated with exacerbating effects in inflammatory processes and autoimmunity (Webb et al., 2016). Besides the activation of co-immunostimulatory receptors by protein-mediated clustering applied in tumor immunotherapy (Fellermeier et al., 2016), the blockade of the ligand-receptor interaction by competing binders is therefore an attractive therapeutic strategy for autoimmune and inflammatory diseases. As demonstrated by Guttman-Yassky et al., 2019, OX40 inhibition by a humanized mAb improved skin gene signatures and clinical scores in patients with atopic dermatitis. Furthermore, the pharmacologic interference with TNF signaling, either through global TNF inhibition, e.g., mediated by anti-TNF antibodies such as infliximab or adalimumab, or more recently through selective blocking of TNFR1 activation has become an accepted therapeutic approach for chronic inflammatory diseases (for review see Fischer et al., 2020).

Anticalin proteins are human-origin binding proteins that have been successfully generated against a variety of targets therapeutically relevant in multiple disease areas (Rothe and Skerra, 2018), e.g. the co-stimulatory checkpoint molecule 4-1BB (Hinner et al., 2019), the IL4-Ra (Matschiner et al., 2018), the extracellular domain of the c-met receptor (Olwill et al., 2013), the Vascular Endothelial Growth Factor A (VEGF-A) (Gille et al., 2016) or the Proprotein Convertase Subtilisin/Kexin type 9 (PCSK9) (Masuda et al., 2018). Anticalin proteins are based on human lipocalins, a family of ubiquitously occurring secretory proteins with transport, storage and scavenger functions. Lipocalins are predestined as scaffolds for binding proteins due to their rigid β-barrel structure in combination with a highly plastic target binding loop region, which translates i.a. into good expression characteristics and high biophysical stability. Anticalin libraries generated by introducing amino acid diversification at certain positions of two lipocalin scaffolds, Neutrophil Gelatinase-Associated Lipocalin (NGAL) or Tear lipocalin (Tlc), can be applied in standard display technologies to select Anticalin proteins with specific binding properties for use in diagnostics or therapy (Skerra, 2008). Due to their small size of ∼20 kDa, Anticalin proteins are rapidly cleared via renal filtration when systemically administered, sharing the fate of most proteins with a mass below ∼60 kDa or a small hydrodynamic radius (Kontermann, 2011). In light of biopharmaceutical development, pharmacokinetic engineering of naked Anticalin proteins can be applied to adjust and extend the inherently short *in vivo* circulation to the specific program need and even reach an antibody-like plasma half-life if necessary.

With respect to a potential application of OX40-directed Anticalin proteins in autoimmune disease therapy based on receptor blockade, alternatives to common IgG- or Fc-based plasma half-life extension strategies that allow a monovalent, non-receptor clustering configuration of Anticalin proteins could be constructive.

One approach for plasma half-life extension in general compatible with this prerequisite is to increase the hydrodynamic radius of small proteins above the threshold of renal filtration. This can for instance be achieved by coupling of hydrophilic polymers such as polyethylene glycol (PEGylation; Milla et al., 2012; Gille et al., 2016) or by fusing repeats of the amino acids Pro, Ala and/or Ser to the protein of interest (PASylation; for review see Gebauer and Skerra, 2018). Moreover, the FcRn-mediated recycling mechanism, enabling an exceptionally long circulation time for the serum proteins albumin and IgG, can be utilized in an indirect manner through fusion of target proteins with 50–60 amino acid residue bacterial binding domains from streptococcal Protein G, such as Albumin-Binding Domain (ABD) and Immunoglobulin-Binding Domain (IgBD). The ABD has earlier been proven as a fusion partner for Anticalin proteins with the aim of immobilizing them in screening assays (Schlehuber et al., 2000). Nowadays, ABDs have been demonstrated to strongly extend the plasma half-life of numerous proteins with otherwise limited pharmacokinetics, including Anticalin proteins (Masuda et al., 2018), molecules based on fibronectin scaffolds (Gapizov et al., 2019), bispecific single-chain diabodies (Stork et al., 2007; Hopp et al., 2010) or affibodies targeting HER2 and HER3 (Andersen et al., 2011; Altai et al., 2018). A deimmunized ABD was derived from an affinity-matured variant that binds Human Serum Albumin (HSA) with a K_D_ in the femtomolar range (Jonsson et al., 2008; Zurdo et al., 2015). In addition to ABDs, IgBDs are suitable to improve the pharmacokinetic profile of small proteins. Although IgBDs have been identified in proteins from a number of bacteria, the C3 domain from Streptococcus Protein G (SpGC3), when fused to a single-chain diabody, showed the strongest effects in terms of plasma half-life extension in mice (Hutt et al., 2012). Naturally, SpGC3 interacts with immunoglobulin G at two distinct sites, one located at CH_1_ in the Fab moiety while the second one is formed by CH_2_ and CH_3_ in the Fc part of IgG (Derrick and Wigley, 1994; DeLano et al., 2000). Due to partly overlapping binding sites, binding of SpGC3 to Fc potentially interferes with the Fc:FcRn interaction which could in consequence lower the efficacy of the endosomal sorting process (Oganesyan et al., 2014). To circumvent this, the specificity of SpGC3 was engineered to bind predominantly the Fab moiety and affinity of IgG binding was restored by phage display maturation to K_D_ values for this binding site within the two-digit nanomolar range (SpGC3FabRR; Unverdorben et al., 2015). As an important prerequisite in view of the endosomal route of FcRn-mediated protein recycling, SpGC3FabRR was shown to form stable interactions with IgG or Fab at neutral as well as slightly acidic pH. This optimized IgBD variant was shown to improve the pharmacokinetics of TRAIL or HER2- and PDGFRβ-targeted bispecific immunotoxins (Yang et al., 2018; Guo et al., 2021).

In the present study, we compared the efficacy of plasma Half-Life Extension (HLE) domains facilitating a monovalent protein configuration, based on fusion of human OX40-specific, receptor blocking Anticalin proteins to ABD and IgBD. We explored the impact of both HLE domains on protein titers, protein bioactivity in SPR and in *in vitro* cell assays and plasma half-life in a wild-type mouse model. More translatable insights into pharmacokinetics were gained from investigating Anticalin-ABD fusion protein in comparison to Anticalin-Fc fusion in a hSA/hFcRn double humanized mouse model. Further, we wanted to explore how the fusion with ABD or IgBD impacts bioactivity and pharmacokinetics of different protein formats, i.e., monospecific Anticalin proteins and larger bispecific Duocalins (Schlehuber and Skerra, 2001), and how affinity or avidity to serum proteins affects pharmacokinetics of this protein class.

## Materials and Methods

### Cell Culture and Materials

HEK293-6E cells were licensed from National Research Council of Canada (Ottawa, Canada) and cultivated in FreeStyle™ F17 Expression Medium (ThermoFisher Scientific) supplemented with GlutaMAX™ (ThermoFisher Scientific, 20 ml/L medium), Kolliphor^®^ P 188 (Sigma-Aldrich, 0.1% w/v) and 25 μg/ml G418 (Enzo, #ALX-380-013) under shaking conditions. Polyethylenimine (PEI; linear, 25 kDa) was purchased from Polysciences GmbH and a 1 mg/ml stock solution was prepared as described (Tom et al., 2008a). Tryptone N1 was from Organotechnie. CHO cells expressing human OX40 and other TNFRSF members were generated by stable transfection of respective expression constructs using the Flp-In System (Invitrogen). NFκB-Luc2/OX40 Jurkat cells (Promega, #J2172) were cultivated in RPMI1640 (Gibco, #61870-010) with 20% FBS (Sigma, #F7524), 1×NEAA (Gibco, #1140-050), 1 mM sodium pyruvate (Gibco, #11360-070), 400 µg/ml hygromycin B (Roth, #CP12.2) and 600 µg/ml geneticin (Promega, #V8091). Flp-In-CHO:huOX40 cells as well as Flp-In-CHO:vector cells generated in-house (Pieris) were cultivated in Ham’s F12 medium (Gibco, #31765-027) with 10% FBS and 500 µg/ml hygromycin B. All mammalian cell lines were grown at 37°C and 5% CO_2_.

Goat anti-rabbit IgG-PE conjugate was purchased from Dianova/Jackson ImmunoResearch (#111-116-144). Flow cytometry antibodies anti-His Tag Alexa Fluor^®^ 488 and Alexa Fluor^®^ 647 anti-human OX40 were from R&D Systems (#IC0501G) and Biolegend (#350018), respectively. Alexa Fluor^®^ 647 Mouse IgG1κ Isotype Control was sourced from BD Biosciences (#557714). Recombinant human OX40L was purchased from Biolegend (#555706). HuIgG1, huIgG2 and huIgG4 for co-incubation in reporter cell assays were from CrownBio (#C0001-3, #C0002-3, C0004-2). HSA was from Sigma (#A3782). OKT3 anti-CD3 antibody and anti-CD28 antibody for co-culture assays were from ThermoFisher Scientific (#16-0037-81) and eBioscience (#MA1-10172), respectively. Human IL-2 was captured and detected with Human IL-2 DuoSet ELISA (R&D Systems, #DY202). Mouse serum (CD-1) was from Innovative Research (#IGMSCD1SER). Mouse plasma (CD-1) was sourced from BioIVT (#MSE00PLLHP2N). Human OX40-Fc was produced in-house by expression of the extracellular domain of human OX40, aa residues 27–214, fused with Fc from huIgG1 at the C-terminus or was purchased from R&D Systems (#3388-OX). Human OX40-His was from Sino Biological (#10481-H08H). SULFO-TAG Streptavidin (#R32AD) and other ECLA specific equipment was from MSD. Anti-His-Tag Antibody (H-3) HRP conjugate was purchased from Santa Cruz (#sc–8036 HRP). Polyclonal rabbit antibodies directed against the NGAL Anticalin scaffold were generated by immunization with a mixture of purified scaffold and Anticalin proteins and affinity chromatography purification of the IgG fraction from serum, followed by biotinylation in-house.

### Construction and Production of Anticalin Proteins

For generation of Anticalin and Duocalin expression constructs, gene cassettes comprising coding sequences for Igκ leader peptide, peptide linkers, HLE modules and 6×His tag were synthesized and cloned into pTT5 vector (Durocher et al., 2002; Tom et al., 2008a; Tom et al., 2008b) to yield acceptor vectors. Anticalin and pre-assembled Duocalin DNA constructs were then cloned into acceptor vectors to achieve combinatorial line-up of constructs. Expression constructs without HLE module or with IgG4 S228P FALA-Fc were generated by cloning of Anticalin or Anticalin-Fc DNA constructs, respectively, into pTT5-ABD acceptor vector under excision of ABD and preceding peptide linker sequences.

For protein production, plasmid DNA preparations were additionally purified with Endotoxin Removal solution (Sigma-Aldrich, #E4274) and HEK293-6E suspension cells were transiently transfected using polyethylenimine as described previously for HEK293-S cells (Siegemund et al., 2018). 24 hours after transfection, 0.5% tryptone N1 was added and cells were grown for another 5 days. Cultures were harvested by centrifugation at 2,000 × *g* for 30 min at 4°C and proteins were purified from supernatants by Ni-NTA IMAC. Supernatants were therefore incubated with PureCube Ni-NTA Agarose (Cube Biotech, #31103, 2 ml bead volume/400 ml supernatant) for 16 h at 4°C on a roller mixer. Beads were collected in an empty column and washed with 7–11 Column Volumes (CV) of IMAC wash buffer (50 mM NaH_2_PO_4_, 300 mM NaCl, 15 mM imidazole, pH 8.0). Bound proteins were eluted with 6 × 0.5 CV of IMAC elution buffer (50 mM NaH_2_PO_4_, 300 mM NaCl, 300 mM imidazole, pH 8.0) followed by dialysis in 1×PBS. Optionally, protein preparations were further purified by preparative gel filtration on a HiLoad 16/600 Superdex^®^ 75 pg or Superdex^®^ 200 increase 10/300 GL column (GE Healthcare). If necessary, eluates were concentrated with Vivaspin^®^ 6 devices, 5 kDa MWCO (Sartorius). Protein concentrations were determined spectrophotometrically using the calculated extinction coefficients. Aliquots were stored at −80 or −20°C.

### Biochemical and Biophysical Protein Analysis

Purified proteins were analyzed by SDS-PAGE under non-reducing and reducing conditions, followed by staining with Coomassie. For size-exclusion chromatography (SEC), a TSKgel^®^ SuperSW mAb HR, 7.8 × 300 mm column (Tosoh) was equilibrated in 0.1 M Na_2_HPO_4_/NaH_2_PO_4_, 0.1 M Na_2_SO_4_, pH 6.7 and proteins were eluted at a flow rate of 0.5 ml/min. Alternatively, SEC was performed on a Zenix-C SEC-300, 4.6 × 150 mM column (Sepax Technologies) in 150 mM sodium phosphate buffer, pH 7.0. The globular proteins thyroglobulin (669 kDa, Rs 8.5 nm), apoferritin (443 kDa, Rs 6.1 nm), β-amylase (200 kDa, Rs 5.4 nm), aldolase (158 kDa), conalbumin (75 kDa), BSA (66 kDa, Rs 3.55 nm), ovalbumin (44 kDa), carbonic anhydrase (29 kDa, Rs 2.35 nm) and RNase A (13.7 kDa) were used as references. Hydrodynamic radii of Anticalin proteins were interpolated after non-linear correlation of SEC retention times with known Rs values of standard proteins.

Midpoints of thermal unfolding (Tm) of Anticalin proteins were determined by Thermal Scanning Fluorimetry (TSF). Therefore, protein samples were mixed at a concentration of 25 µm with 5 µl of 1:100 in 1×PBS diluted SYPRO Orange Protein Gel Stain 5000× stock solution (ThermoFisher Scientific, #S6651) and analyzed in duplicates on a QuantStudio 7 Flex Real-Time PCR System. During measurement, the temperature was ramped from 25 to 95°C with a gradient of 0.015°C/s. Raw data was analyzed with Protein Thermal Shift Software (ThermoFisher Scientific) and TSF Template double read QuantStudio7 v.7 (Pieris).

For Differential Scanning Calorimetry (DSC), the concentrations of Anticalin proteins were adjusted to 1 mg/ml with 1×PBS and measurements were performed on a CSC 6300 Nano-Differential Scanning Calorimeter III (TA Instruments) with 1×PBS as reference, followed by data analysis with NanoAnalyze software (TA Instruments).

For heparin chromatography, 20 µg of each protein was analyzed on a TSKgel^®^ Heparin-5PW HPLC column (0.5 × 5 cm, 10 μm particle size) equilibrated in 20 mM HEPES buffer, pH 7.4. A flow rate of 0.9 ml/min was applied and a linear buffer gradient, which was started 5 min after injection of the sample, was used to establish a buffer condition of 20 mM HEPES, 0.45 M NaCl, pH 7.4 23 min after sample loading.

### Surface Plasmon Resonance

SPR studies were performed on a Biacore T200 instrument (Cytiva). For analysis of OX40 binding by Anticalin proteins, human OX40-His was immobilized at 2.5 µg/ml in 10 mM sodium acetate pH 5.0 on CM5 Series S sensor chips to ∼140 RU using Amine Coupling Kit (Cytiva). HSA (Sigma, #A3782), MSA (Albumin Bioscience), trastuzumab (Roche) and mouse IgG (Sigma, #I5381) were randomly biotinylated using NHS–PEG4–biotin (ThermoFischer Scientific) and immobilized with Biotin CAPture Kit, Series S (Cytiva). Kinetic measurements were performed by injecting serial dilutions of Anticalin proteins in running buffer HBS-EP+, pH 7.4 at a flow rate of 30 µl/min and a temperature of 25°C. For measurements in presence of ABD or IgBD interaction partners, analytes were diluted in HBS-EP+ buffer containing 1.5 µM HSA or trastuzumab. The CM5 sensor chip was regenerated with 10 mM glycine-HCl, pH 2.5 for 60 s. All binding curves were referenced to HBS–EP+ sample and reference flow cell values were subtracted. Binding kinetics parameters were determined using the 1:1 Langmuir binding model or steady state affinity provided by the Biacore T200 Evaluation Software, version 3.0.

### Enzyme-Linked Immunosorbent Assay

For detection of proteins comprising the OX40 Anticalin protein, 96-well high binding plates (Greiner) were coated with OX40-Fc (200 ng/well in PBS) overnight at 4°C. A polyclonal rabbit anti-NGAL antibody (400 ng/well in PBS) was coated the same way for detection of wild-type scaffold proteins. Free binding sites were blocked with assay buffer (2% (w/v) BSA, 0.05% Tween 20 in PBS). Serum samples were diluted in duplicates first 1:10 in assay buffer and further in matrix (assay buffer with 10% CD-1 mouse serum) or directly diluted in matrix for total dilutions of 1:10 or lower to match the detection window of the ELISA with at least 2 dilutions per time point. Purified standard proteins were diluted in matrix and titrated 1:3 in duplicates starting from 100 nM and incubated along with the samples for 2 h at room temperature on the coated plates. Plates were washed three times with washing buffer (0.05% Tween 20 in PBS) after the coating and blocking steps each, respectively, six times after the incubation steps with samples and detection antibody each. For detection of bound proteins, HRP-conjugated anti-His tag antibody was diluted 1:5,000 in assay buffer and incubated for 1 h at room temperature. TMB substrate solution (0.1 mg/ml 3,3′,5,5′-tetramethylbenzidine, 0.02% H_2_O_2_ in 100 mM sodium acetate, pH 6.0) was used for chromogenic development. After having stopped the reaction by addition of 50 µl 1 M H_2_SO_4_, absorption was measured at a wavelength of 450 nm. The limit of quantification was 1–2 ng/ml for both ELISA settings.

### Electrochemiluminescence Assay

For detection of Anticalin proteins in plasma samples by ECLA, 384-well plates (MSD, #L21XA) were coated with OX40-Fc (20 ng/well in PBS) overnight at 4°C, followed by a blocking step with assay buffer (2% (w/v) BSA, 0.05% Tween20 in PBS). After each incubation step, plates were washed five times with 80 µl washing buffer (0.05% Tween20 in PBS). Plasma samples or Anticalin protein standards were diluted in matrix (assay buffer with 10% CD-1 mouse serum for OX40 Ac or with 5% CD-1 mouse serum for OX40 Ac-Fc and OX40 Ac-ABD, respectively) and incubated on the plate in duplicates for 1 h at room temperature under shaking conditions. Bound Anticalin proteins were detected with biotinylated anti-NGAL rabbit polyclonal antibody and SULFO-TAG Streptavidin (0.5 µg/ml) and electrochemiluminescence was measured after addition of 2×MSD Read Buffer T (#R92TC) with a MESO^®^ SECTOR S 600 instrument (MSD). The limit of quantification with this ECLA setting was 0.1–0.2 ng/ml.

For detection of IL-2, 384-well MSD plates were coated with human IL-2 capture antibody (20 ng/well in PBS) for 1 h at 37°C, followed by blocking with 1% casein, 0.1% Tween 20 in PBS at room temperature for at least 1 h. Human IL-2 standard diluted in RPMI1640, 10% FBS, 1% penicillin-streptomycin (Gibco, #15140-122) or cell culture supernatants were then incubated on the plates overnight at 4°C. Bound IL-2 was detected with biotinylated IL-2 detection antibody (50 ng/ml) and SULFO-TAG Streptavidin (1 µg/ml) and electrochemiluminescence was measured as above.

### Flow Cytometry

To investigate binding of Anticalin proteins to OX40 overexpressing cells, Flp-In-CHO:huOX40 cells were harvested and resuspended either in AutoMACS Rinsing Solution (Miltenyi Biotec, #130-091-222) containing 0.5% BSA (Miltenyi Biotec, #130-091-376) or in 1×PBS containing 2% human serum at the density of 5 × 10^4^ cells/well in a 384 well plate, followed by incubation for 1 h on ice. Titrations of the Anticalin proteins starting at 2 µM with a 3-fold dilution series in the same buffers were incubated with the cells for 1 h on ice. Cells were then washed with PBS and incubated with anti-His Tag Alexa Fluor 488-conjugated antibody (1 µg/ml) in either AutoMACS Rinsing Solution, 0.5% BSA or 1×PBS, 2% human serum for 30 min on ice. After a wash step, cells were resuspended in reading buffer (2% Pluronic F-68 (Sigma, # P1300) in 1×PBS) and analyzed on an Intellicyt^®^ iQue Screener PLUS instrument. The expression of OX40 receptor on CHO cells was verified with anti-receptor antibody and isotype control Alexa Fluor^®^ 647 conjugates (data not shown).

### Reporter Cell Assays

To test the OX40 receptor blocking activity of Anticalin proteins, NFκB-Luc2/OX40 Jurkat cells were seeded in RPMI1640, 5% FBS at a density of 1 × 10^4^ cells/well in a 384-well plate (Greiner, #781098). Cells were pre-incubated for 30 min at 37°C and 5% CO_2_ with titrations of the Anticalin proteins starting at 500 nM in a 3-fold dilution series. In case of co-incubation with serum proteins, 1.5 µM HSA or a mixture of huIgG1, huIgG2 and huIgG4 were added to selected concentrations of Anticalin proteins (0.01 nM, 1.95 nM, 500 nM). NFκB-mediated reporter gene expression was then triggered by stimulation with OX40L for 5 h. A ligand concentration of 3 nM was chosen, resulting in a luciferase activity between 50 and 80% of the maximum signal. Plates were then shortly equilibrated to room temperature before Bio Glo Reagent (Promega, #G7940) was added and incubated for 5 min at RT, followed by readout of luminescence with a Cytation 5 instrument (BioTek).

### Co-Culture Assays

Flp-In-CHO:vector cells were treated with mitomycin C (30 µg/ml in PBS; Sigma, # M4287) for 30 min. After washing with PBS, cells were seeded at a density of 8.3 × 10^3^ cells/well on 384-well cell culture plates that were previously coated with 0.25 µg/ml anti-CD3 antibody for 2 h at 37°C. After overnight incubation, supernatants were aspirated and in-house prepared panT cells from human donors, thawed and cultivated overnight, were added at a density of 2.5 × 10^4^ cells/well, followed by a 30 min pre-incubation with titrations of the Anticalin proteins starting at 300 nM in a 3-fold dilution series. T cell reactivity was then stimulated by addition of 4 nM OX40L and 0.05 µg/ml anti-CD28 antibody, followed by further cultivation for 3 days. IL-2 concentration in supernatants was afterwards analyzed by ECLA as described.

### Pharmacokinetics in CD-1 Mice

Animal care and all experiments performed were in accordance with federal and European guidelines and have been approved by the university (animal welfare officer, University of Stuttgart) and state authorities (Regierungspräsidium Stuttgart). Each treatment group consisting of three female, 8–12 weeks old CD-1 mice (Charles River) received a single i.v. bolus injection into the tail vain of approximately 1.2–2.0 mg Anticalin protein per kg bodyweight in a total volume of 100 µl (equals 50 µg of protein). Blood samples were taken 3 min, 1 h, 6 h, 24 h, 72 h and 168 h after administration and immediately incubated on ice. Serum samples were prepared from clotted blood by centrifugation (16,000×*g*, 4°C, 20 min) and stored at −20°C until analysis. The serum concentrations of Anticalin and Duocalin proteins were analyzed by ELISA as described. Data were fitted and interpolated with Prism (GraphPad Software) followed by non-compartmental analysis of pharmacokinetic parameters with PKSolver Add-in for Microsoft Excel (Zhang et al., 2010). Terminal serum half-lives of selected sample pairings were analyzed by Student’s t-test.

### Pharmacokinetics in hSA/hFcRn Double Humanized Mice

This animal experiment, performed at Charles River Discovery Research Services Germany GmbH, was in accordance with federal and European guidelines and has been approved by the state authorities (Regierungspräsidium Freiburg). Each treatment group consisting of two female and one male 6–8 weeks old hSA/hFcRn double humanized mice (C57BL/6N-Fcgrttm1(huFCGRT)Geno Albtm1(huAlb)Geno, Genoway) received a single i.v. bolus injection into the tail vain of 5 mg Anticalin-equivalent per kg bodyweight (equals approximately 4.7 nmol of each protein). Compared to the experiment in CD-1 mice, the dose was here increased to allow a more accurate measurement of plasma concentrations at late timepoints. Blood samples were drawn via microsampling from tail vain and if not applicable retrobulbar. For OX40 Ac, blood samples were taken 3–11 min, 1 h, 3 h, 6 h, 10 h and 24 h after administration. For OX40 Ac-ABD and OX40 Ac-Fc, blood samples were taken 3–25 min, 1 h, 6 h, 24 h, 48 h, 72 h, 96 h and 240 h after administration. Samples were prepared by mixing of blood with a 4-fold volume (OX40 Ac group) or 9-fold volume (OX40 Ac-ABD and OX40 Ac-Fc groups) of Rexxip-A buffer (Gyros Protein Technologies, #P0004820) in ice-chilled Li-Heparin plasma vials, followed by centrifugation and storage of diluted plasma supernatants at −20°C until analysis. The plasma concentrations of the OX40 Anticalin proteins were analyzed by ECLA as described. Data were fitted and interpolated with Excel followed by analysis of pharmacokinetic parameters with the PKSolver Excel Add-in.

## Results

### Construction of Anticalin Fusion Proteins Comprising Monomeric Half-Life Extension Domains

For plasma half-life extension through binding to albumin and IgG we selected the albumin-binding domain Ser–ABD094–Gly (ABD) (Abrahmsén et al., 2012; Frejd, 2012; Zurdo et al., 2015; Masuda et al., 2018) and the immunoglobulin-binding domain SpGC3FabRR (IgBD) (Hutt et al., 2012; Unverdorben et al., 2015) from streptococcal Protein G as fusion partners for Anticalin proteins.

**FIGURE 1:**
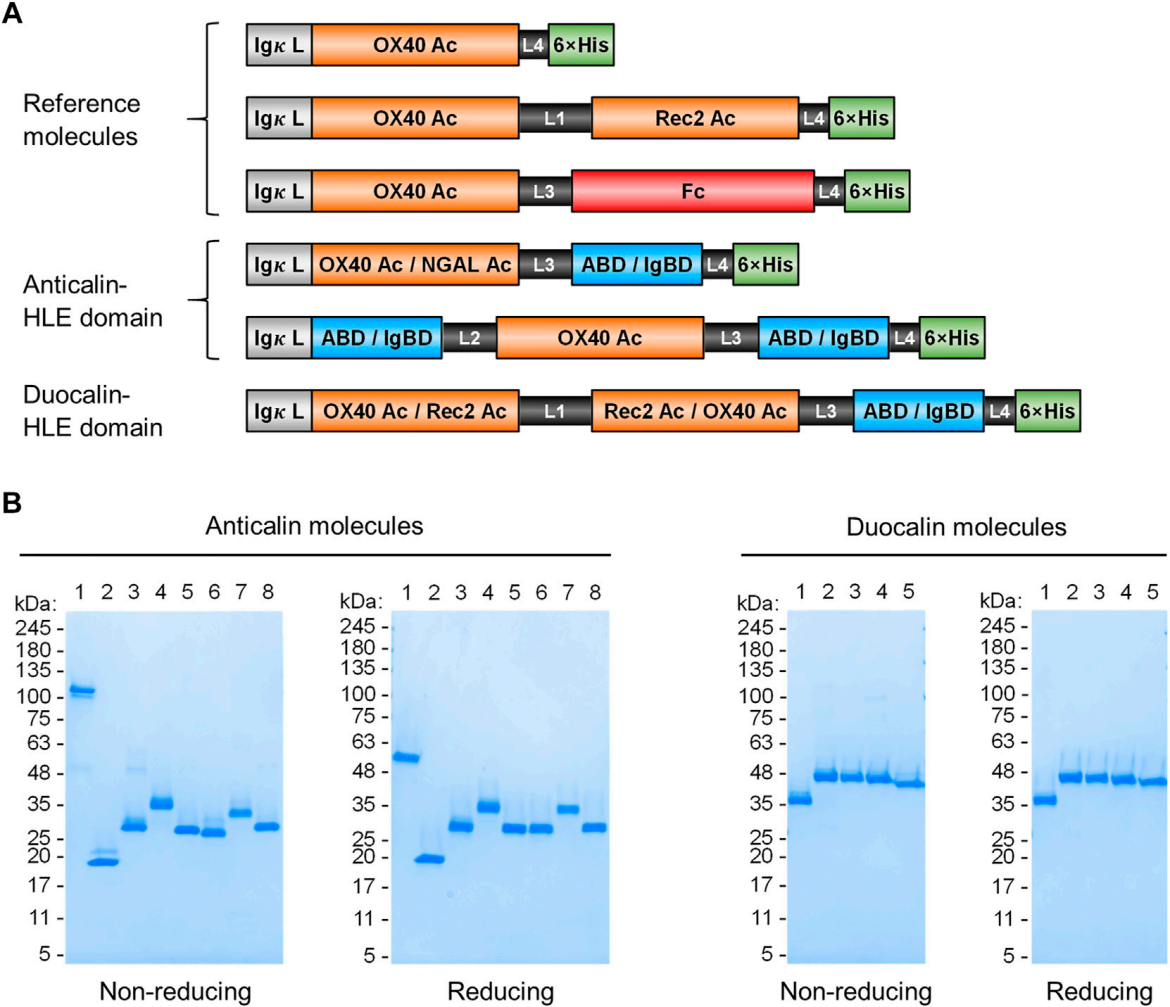
Anticalin proteins fused to serum half-life extension modules. **(A)** Schematic view of the Anticalin fusion proteins generated in this study. An Anticalin protein against OX40 (OX40 Ac), the control NGAL scaffold (NGAL Ac) or an Anticalin protein against another TNFRSF member (Rec2 Ac) were fused with ABD, IgBD or Fc in different Anticalin and Duocalin arrangements. Igκ L, immunoglobulin κ leader peptide; L1, (GGGGS)_3_; L2, GGSGGGGTGG; L3, (GGSGG)_2_; L4, AAAGGS. **(B)** Two µg each of Anticalin proteins **(left)** and Duocalin proteins **(right)** were analyzed by non-reducing and reducing SDS-PAGE and subsequent Coomassie staining. Anticalin proteins: (1) OX40 Ac-Fc, (2) OX40 Ac, (3) OX40 Ac-ABD, (4) ABD-OX40 Ac-ABD, (5) NGAL Ac-ABD, (6) OX40 Ac-IgBD, (7) IgBD-OX40 Ac-IgBD, (8) NGAL Ac-IgBD. Duocalin proteins: (1) OX40 Ac-Rec2 Ac, (2) OX40 Ac-Rec2 Ac-ABD, (3) OX40 Ac-Rec2 Ac-IgBD, (4) Rec2 Ac-OX40 Ac-ABD, (5) Rec2 Ac-OX40 Ac-IgBD.

ABD and IgBD were fused in different arrangements with the Anticalin proteins (Figure 1 A, Table 1). The Anticalin protein binding to human OX40 (OX40 Ac), the parental scaffold protein (NGAL Ac) and a bispecific Duocalin protein composed of two Anticalin proteins were fused at their C-termini either with ABD or IgBD using Gly/Ser peptide linkers with a length of 10 amino acid residues. Duocalin proteins with the capability to antagonize two receptor species were created by joining OX40 Ac via a 15-residue peptide linker with an Anticalin protein directed against a second, non-disclosed co-immunoregulatory TNFRSF member, designated as Rec2 Ac. Two possible orientations of the Anticalin proteins in the Duocalin format were tested. Exemplarily, the ABD and IgBD domains were also fused to the N- and C-terminus of the OX40 Ac, respectively, to assess the influence of two HLE moieties per fusion protein on pharmacokinetics. A fusion of OX40 Ac to a modified human γ4 Fc region (huIgG4 S228P F234A/L235A (FALA)-Fc) (Alegre et al., 1994; Xu et al., 2000; Silva et al., 2015) was chosen as a well-established reference strategy for protein half-life extension.

**TABLE 1:** Biochemical properties of Anticalin proteins.

Molecule	M_r_ (kDa)	R_**S**_ **(nm)**	Yield (mg/L sup.)	T_m_ (°C)
OX40 Ac-Fc	95.7	3.60	67	63
OX40 Ac	21.7	2.32	22.5	66
OX40 Ac-ABD	27.4	2.88	52	61
ABD-OX40 Ac-ABD	33.1	2.82	9	62
NGAL Ac-ABD	27.4	2.77	113	64
OX40 Ac-IgBD	28.4	2.60	8.7	64
IgBD-OX40 Ac-IgBD	35.1	2.94	3.4	—
NGAL Ac-IgBD	28.4	2.52	10	66
OX40 Ac-Rec2 Ac	39.3	2.91	23	63
OX40 Ac-Rec2 Ac-ABD	45.0	3.70	54	63
OX40 Ac-Rec2 Ac-IgBD	46.0	3.34	16	62
Rec2 Ac-OX40 Ac-ABD	45.0	3.42	48	61
Rec2 Ac-OX40 Ac-IgBD	46.0	3.32	18	61

—, not determined.

Upon recombinant expression and subsequent IMAC purification, we observed yields between 48–113 mg/L for ABD and ∼9–18 mg/L for IgBD C-terminal fusions (Table 1). Molecules with N- and C-terminal fusion of ABD and IgBD showed yields of 9 mg/L and 3.4 mg/L, respectively. All variants yielded soluble protein without impurities or degradation products, composed of full-length polypeptide chains matching the expected molecular masses as proven by non-reducing and reducing SDS-PAGE (Figure 1B). The dimerization of the OX40 Ac-Fc fusion was confirmed under non-reducing conditions. Importantly, all protein preparations from IMAC showed a high homogeneity in Size-Exclusion Chromatography (SEC) with a consistently low presence of higher MW forms (Supplemental Figure S1). Additional preparative SEC was conducted for a subset of proteins to ensure a monomer content of ≥95% for all samples. In summary, from the aspect of protein production, both ABD and IgBD proved as fusion partners for Anticalin proteins with yield benefits in case of ABD.

### Biochemical Characterization of Anticalin Proteins

Thermal stability measurements of the Anticalin proteins were performed by thermal scanning fluorimetry. Assuming a convolution of overlapping unfolding transitions of Anticalin and HLE components, fusion of OX40 Ac to ABD and IgBD led to a reduction of Tm of 5 and 2°C compared with the naked Anticalin protein, respectively (Table 1). The fusion of the Anticalin protein to Fc resulted in a 3°C reduction of Tm. In a more precise experimental setup using differential scanning calorimetry we analyzed scaffold NGAL Ac-ABD and NGAL Ac-IgBD referenced to NGAL Ac without HLE module and could confirm a higher thermostability of NGAL Ac-IgBD compared to NGAL Ac-ABD, the latter showing a thermal unfolding very similar to the NGAL Ac without HLE module (Supplemental Figure S2).

Next, we applied analytical SEC to test if OX40 Ac-ABD and -IgBD fusion proteins form complexes with human and mouse serum albumin or IgG, respectively. Incubation and subsequent analysis of equimolar amounts of OX40 Ac-ABD with HSA and MSA resulted in quantitative complex formation, indicated by earlier elution of the complex peaks and disappearance of the OX40 Ac-ABD peaks in the mixture, whereas incubation with naked OX40 Ac had no effect (Supplemental Figure S3). In contrast, besides a weak effect in combination with mouse IgG, we did not observe binding of OX40 Ac-IgBD to IgG using this method, leading to the assumption that IgG is much weaker bound by the IgBD compared with the binding of serum albumins by the ABD.

We analyzed Anticalin proteins by heparin chromatography because of its predictive character for clearance of proteins from circulation mediated by pinocytosis. Heparin solid phases have been described as an *in vitro* surrogate for highly negatively charged glycocalyx components on endothelial cells (Kraft et al., 2020). Accordingly, enhanced retention times of positively charged proteins in heparin chromatography have been shown to correlate with nonspecific cell surface interaction and faster clearance. Though, we observed short retention times in the range of the negative control panitumumab, suggesting a low impact of nonspecific clearance on pharmacokinetics for the Anticalin fusion proteins (Supplemental Table S1).

### Binding Activity of Anticalin Proteins

**FIGURE 2:**
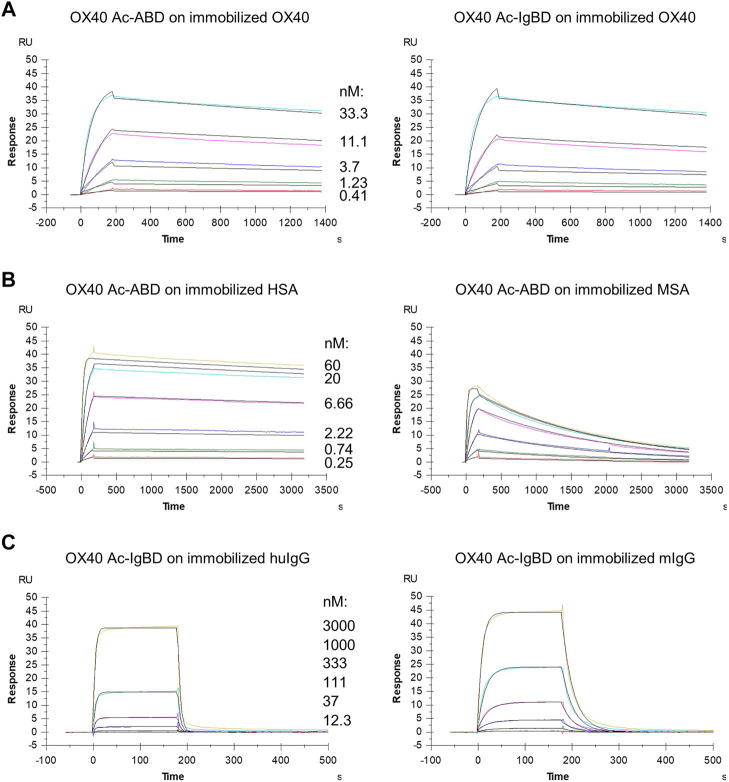
Binding of Anticalin fusion proteins to OX40 receptor and serum proteins in SPR. The sensorgrams show the binding kinetics of exemplary OX40 Ac-ABD and OX40 Ac-IgBD analytes applied in serial dilutions to the immobilized OX40 receptor or serum protein ligands. Black lines represent a 1:1 model fit. **(A)** OX40 Ac-ABD **(left)** and OX40 Ac-IgBD **(right)** were analyzed for binding to recombinant huOX40-His coupled to a CM5 sensor chip. **(B)** OX40 Ac-ABD was analyzed for binding to biotinylated HSA **(left)** and biotinylated MSA **(right)** ligands immobilized on a CAP sensor chip. **(C)** OX40 Ac-IgBD was analyzed for binding to biotinylated trastuzumab **(left)** and biotinylated mouse IgG **(right)** ligands immobilized on a CAP sensor chip.

We wanted to investigate whether the fusion to ABD and IgBD per se or the additional presence of the binding partners HSA or huIgG has an impact on the binding activity of the Anticalin proteins to OX40. Thus, we analyzed the binding of Anticalin proteins to immobilized OX40 receptor in SPR with and without the presence of 1.5 µM HSA or trastuzumab, the latter chosen as a model for huIgG. The C-terminal or dual N- and C-terminal fusion of either ABD or IgBD to the OX40-specific Anticalin and Duocalin proteins alone did not influence the affinity for OX40, as demonstrated by retained sub-nanomolar K_D_ values without exception (Figure 2A, Table 2). In presence of HSA we observed however ∼2–2.5-fold increased K_D_ values for OX40 Ac fusions with single ABD, whereas dual ABD fusions reduced the affinity for OX40 up to 4-fold. Similarly, the presence of trastuzumab led to ∼1.7–2-fold increased K_D_ values for single IgBD fusions and a ∼10-fold increase of K_D_ in case of IgBD-OX40 Ac-IgBD.

**TABLE 2:** K_D_ values of Anticalin proteins binding to OX40 receptor or serum proteins as determined by SPR.

	OX40 binding			SA binding		IgG binding	
Molecule	K_D_ (×10^−9^ M)	+HSA; K_D_ (×10^−9^ M)	+huIgG^a^; K_D_ (×10^−9^ M)	HSA; K_D_ (×10^−9^ M)	MSA; K_D_ (×10^−9^ M)	huIgG^a^; K_D_ (×10^−6^ M)	muIgG; K_D_ (×10^−6^ M)
OX40 Ac-Fc	0.14	0.35	—	—	—	—	—
OX40 Ac	0.40	0.48	0.46	n. b.	n. b.	n. b.	n. b.
OX40 Ac-ABD	0.29	0.70	0.40	0.043	0.41	n. b.	n. b.
ABD-OX40 Ac-ABD	0.37	1.44	0.42	0.071	0.66	—	—
NGAL Ac-ABD	n. b.	—	—	0.054	0.69	n. b.	n. b.
OX40Ac-IgBD	0.42	0.50	0.84	n. b.	n. b.	10.1^b^	1.83
IgBD-OX40 Ac-IgBD	0.48	0.51	4.9	—	—	0.12	0.013
NGAL Ac-IgBD	n. b.	—	—	n. b.	n. b.	2.24^b^	0.71
OX40 Ac-Rec2 Ac	0.74	0.76	0.78	—	—	—	—
OX40 Ac-Rec2 Ac-ABD	0.57	1.13	0.58	0.058	0.58	—	—
OX40 Ac-Rec2 Ac-IgBD	0.80	0.78	1.49	—	—	11.8^b^	1.83
Rec2 Ac-OX40 Ac-ABD	0.60	1.31	0.62	0.081	0.94	—	—
Rec2 Ac-OX40 Ac-IgBD	0.74	0.75	1.26	—	—	14.9^b^	2.61

a Trastuzumab

b Steady state affinity

—, not determined; n. b., no binding detected

Next, we analyzed the binding of Anticalin-ABD and -IgBD fusions to serum albumin and IgG from human and mouse, respectively (Figure 2B,C, Table 2). We observed the highest affinity for binding of Anticalin-ABD proteins to HSA with K_D_ values in the two-digit picomolar range (0.043–0.081 nM), whereas binding to MSA was approximately 10-fold weaker (K_D_ = 0.41–0.94 nM). In contrast, the affinity of Anticalin-IgBD proteins to trastuzumab was up to ∼235,000-fold weaker in comparison to the binding of Anticalin-ABD fusions to HSA. Due to very fast on- and off-rates, steady state affinities for these interactions were determined with K_D_ values in the micromolar range, reaching from 2.24–14.9 µM for IgBD single fusions. Related to that, mouse IgG was bound stronger with K_D_ values in the range from 0.71–2.61 µM. Potentially in consequence of an avidity effect due to the presence of two Fab moieties per IgG molecule, IgBD-OX40 Ac-IgBD showed approximately 100-fold increased affinity to IgG, which was not observed for binding of ABD-OX40 Ac-ABD to serum albumins.

**FIGURE 3:**
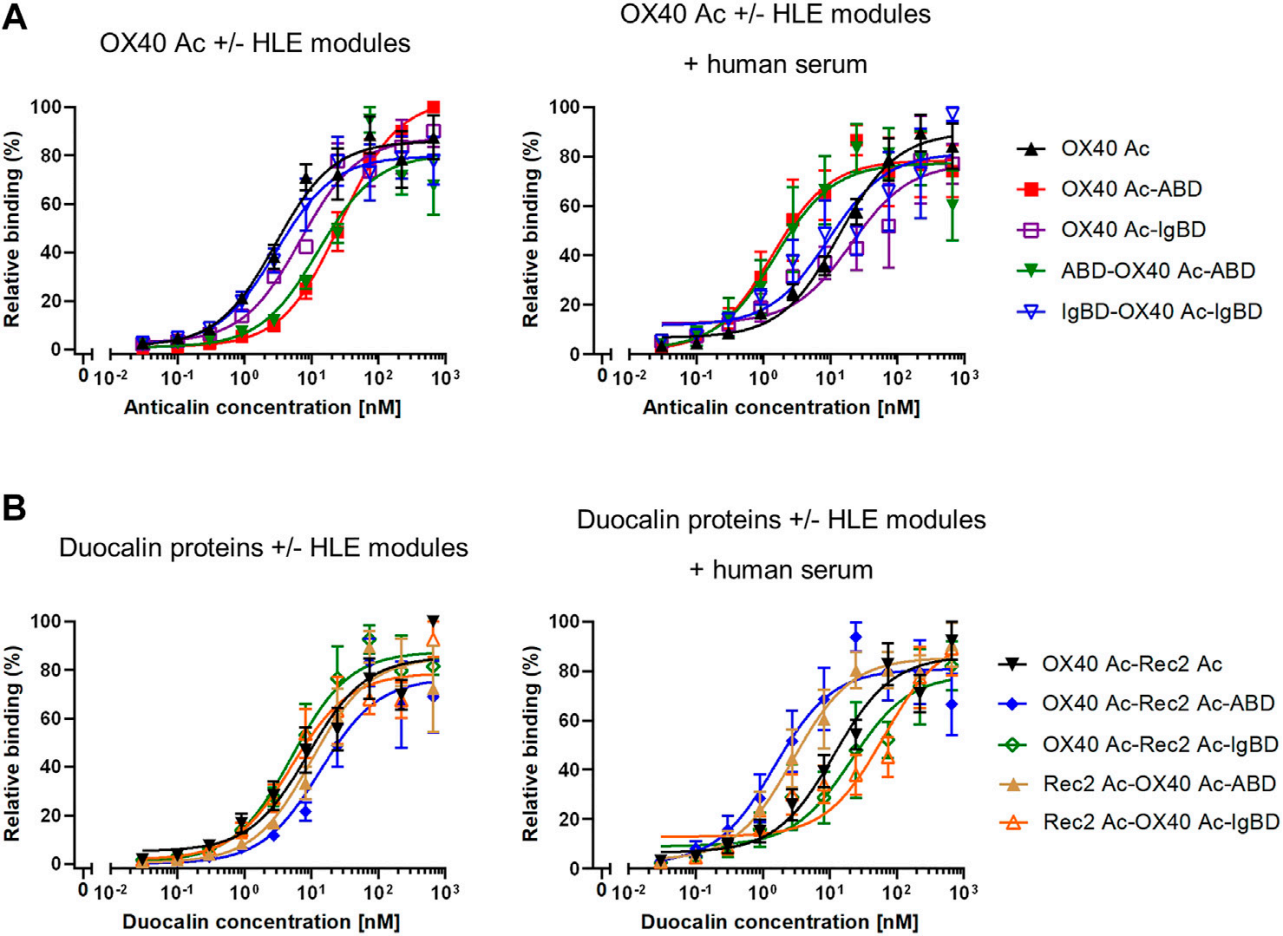
Binding of Anticalin proteins to OX40 receptor expressed on CHO cells. **(A)** OX40-specific Anticalin proteins or **(B)** Duocalin proteins specific for OX40 and a second TNFRSF receptor were analyzed for binding to Flp-In-CHO:huOX40 cells in flow cytometry in absence **(left diagrams)** or presence of 2% human serum **(right diagrams)**. Normalization of data was done by setting the highest fluorescence value in each sample titration row to 100% and the fluorescence of the cell control to 0%. Data is represented as mean ± S.E.M. (standard error of the mean) of normalized datasets from four experiments.

In cell-based binding assays, OX40 Anticalin-ABD and -IgBD fusion proteins bound to OX40-overexpressing Flp-In-CHO cells in a manner almost comparable to naked OX40 Ac, which was also seen for the corresponding Duocalin protein formats (Figure 3, Supplemental Table S2). However, in the presence of human serum that potentially enables complex formation with HSA and huIgG, cell binding of OX40-specific IgBD fusion proteins was lower, whereas cognate ABD fusions showed some improved binding. In addition, the experiment was performed with NGAL scaffold-ABD and -IgBD fusion proteins on Flp-In-CHO:huOX40 cells as well as with OX40-specific Anticalin proteins on Flp-In-CHO:vector cells as negative controls showing no unspecific binding in both cases (data not shown). An expression control of Flp-In-CHO:huOX40 cells was performed in every experiment. In summary, Anticalin-ABD fusions were superior to Anticalin-IgBD fusions in terms of binding to target serum proteins. For the OX40-specific ABD and IgBD fusion molecules, the binding activity towards OX40 receptor was differently influenced by the presence of the respective target serum proteins depending on the assay.

### Receptor Blocking Bioactivity of Anticalin Proteins on Cells

**FIGURE 4:**
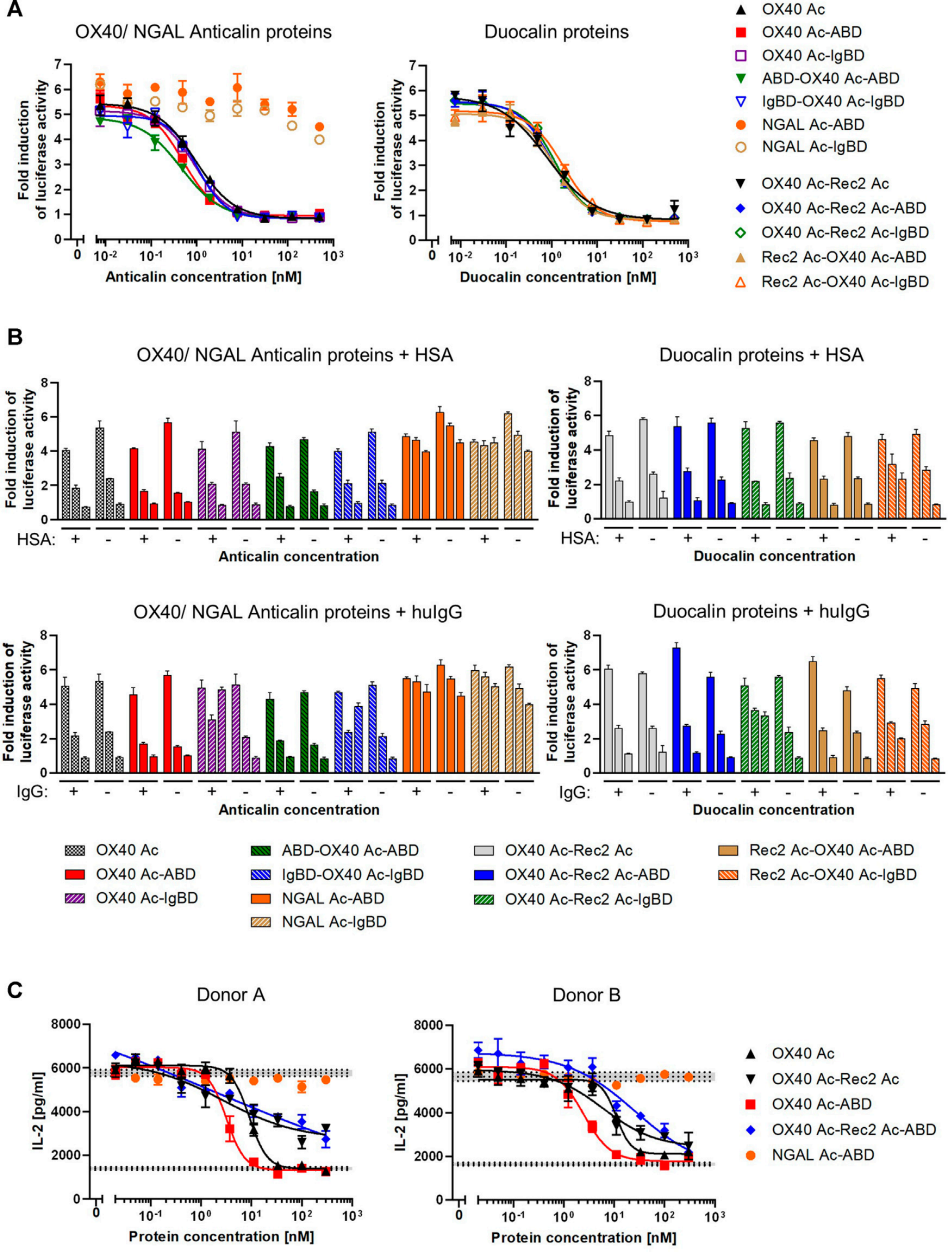
Functional blocking of OX40 receptor by Anticalin proteins *in vitro*. **(A)** OX40 antagonizing Anticalin proteins were added in titration to NFκB-Luc2/OX40 Jurkat reporter cells 30 min before stimulation with 3 nM OX40 ligand. The ligand concentration was previously adjusted to result in a NFκB-controlled luciferase activity approximately in the range of a half-maximal effect. After 5 h of ligand stimulation, luciferase substrate was added and resulting luminescence was measured. Data is represented as mean ± S.E.M. of triplicate values from one experiment, which is representative of three independent experiments. **(B)** Luciferase activity was monitored after addition of 1.5 µM HSA **(upper graphs)** or a mixture of equimolar amounts of human IgG1 IgG2 and IgG4 **(lower graphs)** in parallel with the Anticalin or Duocalin proteins. Column triplets represent three selected concentrations of Anticalin proteins (from left to right: 0.01, 1.95, 500 nM). Data is represented as mean ± S.E.M. of triplicate values. This data is a representation from three independent experiments. **(C)** The OX40 receptor blocking activity of a subset of Anticalin or Duocalin proteins was assessed in a co-culture assay setting. Therefore, panT cells from donor A or B were cultivated at a ratio 3:1 with mitomycin C treated Flp-In-CHO:vector cells to stimulate T cell alloreactivity. Four nM OX40 ligand was added to stimulate T cell activation in presence of anti-CD3 and anti-CD28 antibody. Dose-dependent inhibition of IL-2 release by OX40-specific Anticalin proteins is shown. Data is represented as mean ± S.E.M. of triplicate values from one experiment. Middle horizontal dotted lines represent the average of the background signal with anti-CD3 antibody, Flp-In-CHO:vector, panT cells and anti-CD28 antibody either in presence of 4 nM of OX40L (upper level of activation) or in absence of the ligand (lower background). Gray areas represent standard error of the mean of the average value for respective background levels.

To assess competition of Anticalin proteins with natural OX40 ligand for receptor binding, we applied a luciferase reporter cell assay with NFκB-Luc2/OX40 Jurkat cells overexpressing OX40 receptor on their surface. OX40 receptor blocking by specific Anticalin proteins prevents OX40 ligand triggered activation of the NFκB signaling pathway and reduces the luciferase signal. Upon preincubation of reporter cells with titrations of OX40-specific Anticalin proteins and subsequent stimulation with a constant concentration of OX40 ligand we observed concentration-dependent inhibition of the reporter signal with IC_50_ values between ∼0.5 and 1 nM. Scaffold Anticalin fusions showed no blocking activity as expected (Figure 4A, Supplemental Table S3). OX40-specific Duocalin proteins exerted bioactivity comparable to Anticalin proteins in the reporter cell assay along with a similarly low impact of the protein format or HLE domain on OX40 inhibition. Analogously to the previous binding studies, we analyzed the inhibitory activity of Anticalin proteins also in a separate setting using co-incubation of the proteins with HSA and huIgG (Figure 4B). At the three tested Anticalin protein concentrations, addition of HSA did not explicitly result in an attenuation of the receptor blocking activity by OX40-specific Anticalin- and Duocalin-ABD fusion proteins. However, co-incubation with huIgG was shown to inhibit the bioactivity of OX40-specific Anticalin-IgBD fusions and to a lesser extent of Duocalin-IgBD fusions.

In another setting, we used panT cells from human donors which were stimulated by co-culture with Flp-In-CHO cells. The blocking of OX40 receptors by anti-OX40 Anticalin proteins resulted in an inhibition of the T cell response as measured by a decreased IL-2 release upon stimulation with a combination of the external factors anti-CD3 antibody, anti-CD28 antibody and OX40 ligand. In this more physiological assay we observed in contrast to the reporter assay a pronounced impact of the protein format on the blocking activity of OX40-specific Anticalin proteins (Figure 4C, Supplemental Table S4). By taking inhibition of T cell responses for four different donors into account, OX40 Ac-ABD was slightly more effective compared to the naked Anticalin protein. Considering variation between donors, OX40-specific Duocalin proteins showed a somewhat lower potency of T cell inhibition compared to Anticalin proteins, as shown by flattened dose-response curves and incomplete inhibition at higher concentrations.

Overall, we confirmed that OX40-specific binding of Anticalin proteins as well as Anticalin HLE domain fusion proteins, as already determined by SPR and flow cytometry, did translate into functional blocking of OX40 ligand-mediated receptor signaling and reduced T cell responses.

### Mouse Pharmacokinetics of Anticalin Proteins

**FIGURE 5:**
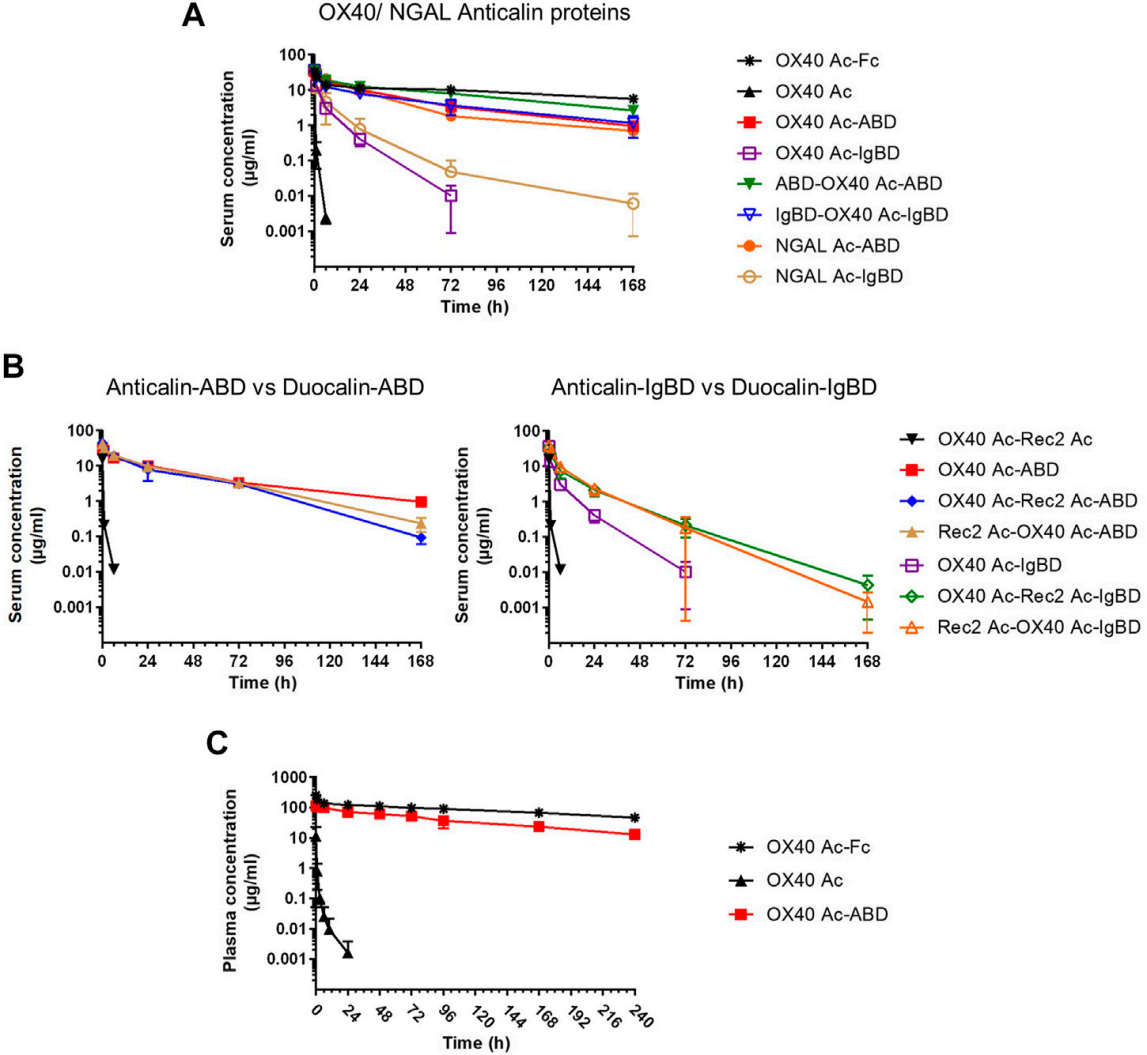
Pharmacokinetics of Anticalin proteins in mice. **(A, B)** The serum concentrations after i.v. administration of 1.2–2.0 mg per kg bodyweight of Anticalin or Duocalin proteins with or without HLE module in CD-1 mice were analyzed by ELISA (n = 3, mean ± S.D.). **(A)** Comparison of serum concentrations over time for OX40 or NGAL Anticalin proteins. **(B)** The serum concentrations of corresponding Duocalin proteins were plotted against the PK of OX40 Ac-ABD **(left)** or OX40 Ac-IgBD **(right)**. **(C)** The plasma concentrations after i.v. administration of 5 mg/kg Anticalin equivalent of OX40 Ac, OX40 Ac-ABD and OX40 Ac-Fc in hSA/hFcRn double humanized mice were analyzed by ECLA (n = 3, mean ± S.D.).

We investigated the pharmacokinetics of the Anticalin-ABD and -IgBD fusion proteins in CD-1 wild-type mice using OX40 Ac-Fc and naked OX40 Ac as references. The fusion of the human-specific OX40 Ac with ABD and IgBD did substantially extend its terminal serum half-life (*t*_1/2_ = 40.3 and 7.2 h) in relation to the naked Anticalin protein (*t*_1/2_ = 0.55 h). OX40 Ac-Fc showed however the longest presence in the circulation with a *t*_1/2_ of 130 h (Figure 5A, Table 3). This general efficacy of half-life extension (Fc > ABD > IgBD) was consistent between the NGAL-based Anticalin proteins and Anticalin proteins based on a tear lipocalin scaffold including Rec2 Ac and Tlc Ac (data not shown). Interestingly, we observed an improved PK profile of proteins with dual (N- and C-terminal) configuration of the HLE domains compared to the single domain fusions. The *t*_1/2_ of IgBD-OX40 Ac-IgBD was almost 7-fold longer in relation to OX40 Ac-IgBD (50 vs. 7.2 h). ABD-OX40 Ac-ABD showed a ∼1.5-fold prolonged *t*_1/2_ compared to OX40 Ac-ABD (62.6 vs. 40.3 h).

**TABLE 3:** Pharmacokinetic parameters of Anticalin proteins in CD-1 wild-type mice (n = 3, mean ± S.D.).

Molecule	Cl (ml/h/kg)	AUC_0-inf_ (μg/ml*h)	Terminal *t*_1/2_ (h)
OX40 Ac-Fc	0.65 ± 0.11	2,676 ± 204	130 ± 9
OX40 Ac	227 ± 26	7.6 ± 0.9	0.55 ± 0.03
OX40 Ac-ABD	1.43 ± 0.08	974 ± 84	40.3 ± 1.8
ABD-OX40 Ac-ABD	1.10 ± 0.04	1,677 ± 67	62.6 ± 2.5
NGAL Ac-ABD	1.68 ± 0.02	867 ± 58	31.2 ± 3.4
OX40 Ac-IgBD	12.6 ± 0.6	107 ± 5	7.2 ± 2.2
IgBD-OX40 Ac-IgBD	1.91 ± 0.41	898 ± 237	50.0 ± 11.5
NGAL Ac-IgBD	14.4 ± 5.7	144 ± 75	15.0 ± 4.2
OX40 Ac-Rec2 Ac	195 ± 19	9.0 ± 1.0	0.69 ± 0.02
OX40 Ac-Rec2 Ac-ABD	2.10 ± 0.37	826 ± 154	21.2 ± 2.1
OX40 Ac-Rec2 Ac-IgBD	6.5 ± 1.5	256 ± 59	15.1 ± 2.2
Rec2 Ac-OX40 Ac-ABD	1.83 ± 0.20	922 ± 156	25.9 ± 1.8
Rec2 Ac-OX40 Ac-IgBD	6.4 ± 1.2	293 ± 56	12.3 ± 1.8

In combination with the larger Duocalins (Figure 5B, Table 3) we observed also a clear pharmacokinetic benefit of ABD (*t*_1/2_ = 21.2 and 25.9 h) and IgBD (*t*_1/2_ = 15.1 and 12.3 h) fusion proteins compared to the naked Duocalin (*t*_1/2_ = 0.69). The ABD showed again the stronger effect on serum half-life extension although the differences between Duocalin-ABD and -IgBD fusions were smaller compared to the Anticalin counterparts. The *t*_1/2_ of the Duocalin-IgBD fusion proteins were longer compared to OX40 Ac-IgBD (15.1 and 12.3 h vs. 7.2 h), which was moreover reflected in at least ∼2-fold higher AUC values. At this point it should also be noted that *t*_1/2_ of Rec2 Ac-IgBD was similarly short with 4.3 ± 0.7 h (data not shown). In contrast, for Duocalin-ABD fusion proteins we observed shorter *t*_1/2_ in relation to the respective Anticalin-ABD variants (21.2 and 25.9 h vs. 40.3 h for OX40 Ac-ABD and 42.0 ± 1.3 h for Rec2 Ac-ABD; data not shown for the latter), though with all AUC values in a similar range. The orientation of Anticalin units within the Duocalin molecule did however not result in a statistically significant influence on terminal serum half-life as determined by *t*-test (p = 0.077, Duocalin-ABD; p = 0.24, Duocalin-IgBD). In general, we would exclude an influence of on-target effects on PK due to lacking cross-reactivity of OX40 Ac and Rec2 Ac towards their orthologous mouse targets.

To obtain PK data with enhanced translatability, we analyzed OX40 Ac, OX40 Ac-ABD and OX40 Ac-Fc in a hSA/hFcRn double humanized mouse model (Viuff et al., 2016; Figure 5C, Table 4). In this model, both Anticalin-ABD and -Fc fusion proteins can interact with human serum albumin and human FcRn, respectively, which facilitates a more accurate prediction of the PK in humans. While *t*_1/2_ of the naked OX40 Ac was also in this model short (4.3 h), we observed a prolonged mean *t*_1/2_ for OX40 Ac-ABD in comparison to the CD-1 model (110 vs. 40.3 h). This correlates with the longer half-life of HSA in humans compared to MSA in mice (Dixon et al., 1953). The *t*_1/2_ of OX40 Ac-Fc in the hSA/hFcRn model was similar to the CD-1 model (146 vs. 130 h). The ∼2.5–3-fold prolonged terminal plasma half-life of OX40 Ac-ABD in hSA/hFcRn mice compared to CD-1 mice was also confirmed for Rec2 Ac-ABD (*t*_1/2_ = 111 ± 28 h; data not shown).

**TABLE 4:** Pharmacokinetic parameters of Anticalin proteins in hSA/hFcRn mice (n = 3, mean ± S.D.).

Molecule	Cl (ml/h/kg)	AUC_0-inf_ (μg/ml*h)	Terminal *t*_1/2_ (h)
OX40 Ac-Fc	0.37 ± 0.09	30,759 ± 3,985	146 ± 21
OX40 Ac	935 ± 473	8.1 ± 5.5	4.3 ± 0.3
OX40 Ac-ABD	0.53 ± 0.00	11,686 ± 996	110 ± 31

In summary, our PK data of Anticalin fusion proteins point clearly to a positive correlation between the affinity to the FcRn-recycled serum protein and the resulting plasma half-life *in vivo*. Anticalin and Duocalin formats showed differential pharmacokinetic properties when C-terminally fused with either ABD or IgBD.

## Discussion

Pharmacokinetics of small biologics and their retention time in plasma can be improved by various means, e.g., by simple increase of the hydrodynamic radius or active strategies involving FcRn-mediated protein recycling (Kontermann, 2011). Moreover, a balance between therapeutic efficacy, patient-friendly administration schedules and the reduction of adverse events might be established by choosing the right HLE approach for a biopharmaceutical candidate during development. Our present work focused on new candidates with potential for treatment of autoimmune diseases. We developed a concept to endow small, OX40 receptor-antagonizing Anticalin proteins with streptococcus-derived albumin or Ig binding domains to realize plasma HLE in combination with a monovalent protein format. The latter circumvents potential receptor dimerization and therefore activation which might otherwise be promoted in consequence of fusion to dimeric Fc or IgG. The chosen approach enabled the generation of per se small, monospecific Anticalin and bispecific Duocalin fusion proteins with molecular masses of ∼28 and ∼45 kDa, respectively, exhibiting prolonged plasma half-life.

The OX40:OX40L interaction is of relevance in autoimmunity and its blockade has for example been demonstrated to ameliorate disease symptoms and restore tissue integrity in rheumatoid arthritis (Gwyer Findlay et al., 2014). Particularly the Duocalin fusion protein format could be a promising strategy to interfere with dysregulated processes in autoimmunity because the simultaneous blocking of two co-immunoregulatory receptors is supposed to increase therapeutic benefits. Notably, the bispecific Duocalin proteins fulfil similarly to Anticalin proteins the prerequisite of monovalency regarding each receptor target.

Regarding functional OX40 receptor blocking in a co-culture T cell activation assay, differences between the Anticalin and the Duocalin format were detected. A possible explanation is provided by the presence of the Rec2-blocking Anticalin in the Duocalin molecules which could additionally modulate panT cell responses with effect on total IL-2 release. Other specific properties like slightly lower on-rates for OX40 binding in SPR (data not shown) could also potentially interfere with the bioactivity of the Duocalin fusion proteins. Regarding receptor-dependent functionality, future experiments could reveal the precise mechanism, how OX40-specific Anticalin proteins exert their inhibiting activity on T cells, particularly in view of the T cell subsets.

Our functional data further show that OX40 receptor binding by Anticalin-ABD or -IgBD proteins is not affected by the fusion with the small HLE domains per se. The presence of human serum or IgG in OX40-specific cellular binding and receptor blocking assays did however result in a negative effect on bioactivity of OX40 Ac comprising IgBD fusions, which was not observed in similar settings with corresponding ABD fusions and human serum/albumin. It could be hypothesized that the larger size of IgG or a more unfavorable steric geometry of the OX40 Ac-IgBD:IgG complexes contributes to this observation. The use of surrogate proteins in mouse models of autoimmune diseases might shed light on the relevance of this effect *in vivo*, also in view of lower IgG concentrations in interstitial spaces of tissues compared to blood (Eigenmann et al., 2017).

As a general prerequisite, a biotherapeutic must reach effective concentrations in target tissues. In this regard, a linear correlation between tissue levels and plasma concentrations has been shown for antibody fragments (Li et al., 2016). Li et al. have also demonstrated that biodistribution coefficients and thus tissue concentrations of proteins decrease exponentially with increasing protein size, suggesting that small therapeutic proteins are generally preferable over large, e.g. IgG-like formats in terms of tissue penetration. The Anticalin-ABD and -IgBD fusion proteins with their hydrodynamic radii between 2.6 and 3.7 nm and thus smaller size than IgG (5.41 nm; Armstrong et al., 2004) fit well into this concept of a small protein with FcRn-mediated plasma half-life, being suitable to reach sufficient tissue concentrations. However, the additional contribution of the non-covalently bound serum protein partner albumin or IgG to the total molecule size and bioactivity must be taken into consideration. Further biodistribution studies either *in vivo* or with tissue models could reveal the diffusion properties of such protein complexes and could help to clarify, whether Anticalin-ABD or -IgBD fusion proteins in complex with blood-derived serum proteins are potentially able to diffuse freely within tissues.

Interestingly, albumin has been shown to accumulate at tumors and sites of inflammation where it is metabolized to cover the increased need of activated cells for amino acids and energy (reviewed in Neumann et al., 2010). Possibly, this accumulation of albumin can even promote the targeting of inflamed tissues by Anticalin-ABD:albumin complexes as already demonstrated for albumin-based delivery of drugs in a mouse model of rheumatoid arthritis (Wunder et al., 2003). On the contrary, the targeting of IgG for modulation of serum half-life as demonstrated for Anticalin-IgBD proteins could be favored in terms of physiological aspects associated with IgG and its FcRn-mediated recycling. The homeostasis of IgG in human plasma is predominantly controlled by the recycling activity of FcRn, whereas plasma albumin concentration is maintained rather by *de novo* protein synthesis (Kim et al., 2007). The absolute synthesis rate of about 150 mg/kg/day in healthy humans indicates that albumin turnover is indeed far more rapid than the average body protein (Levitt and Levitt, 2016). IgG is therefore in principle the more attractive target for plasma half-life extension, also in consideration of its somewhat longer plasma half-life compared to albumin in humans (up to ∼21 vs. ∼17 days; Mankarious et al., 1988; Levitt and Levitt, 2016).

Beside these physiological considerations, also biophysical characteristics of the HLE modules, e.g. affinity to serum proteins, influence their efficacy. Our data from SEC experiments and SPR binding studies show that the affinity of the ABD for albumin is magnitudes higher than the affinity of the IgBD for IgG, which provides a reasonable explanation for the superiority of the ABD in terms of plasma HLE. It has been shown for albumin-binding domains that a higher affinity to albumin correlates with better pharmacokinetic properties (Hopp et al., 2010; Jacobs et al., 2015), which suggests a similar relationship for the IgBD:IgG system. In this regard, an approximately 7-fold extension of terminal plasma half-life was achieved by N- and C-terminal fusion of the IgBD to the OX40 Ac compared to only C-terminal fusion. This beneficial effect of two HLE modules was less pronounced in the corresponding molecule with dual ABD. Moreover, this observation correlates with the ∼100-fold improved binding to IgG of the dual IgBD fusion compared to the IgBD monofusion. Here, one could hypothesize an avidity effect mediated through simultaneous binding of both IgBD domains from IgBD-OX40 Ac-IgBD to the two CH1 domains per IgG molecule. Future studies, e.g., by SEC-MALS, could reveal the actual stoichiometries of IgBD-Anticalin-IgBD:IgG and ABD-Anticalin-ABD:albumin complexes, also in view of their pharmacokinetic impact. Nevertheless, a dual HLE domain format for Anticalin IgBD fusion proteins might to some extent overcome the pharmacokinetic limitations attributable to the rather low affinity of the IgBD to IgG. Besides the affinity to the recycled serum protein as a clear determinant for plasma half-life of Anticalin fusion proteins, our PK data from Duocalin proteins suggest an additional influence of the Anticalin protein format or size, leading to different outcomes for ABD and IgBD. The PK properties of IgBD fusions seem to profit from the Duocalin format rather than from the Anticalin format. This effect was in terms of terminal plasma half-life reversed for ABD fusions, albeit the here observed general principle of plasma half-life extension efficacy “Fc > ABD > IgBD” was not affected. In this regard, Duocalin-specific properties like differing biophysical parameters (e.g., hydrophobicity/hydrophilicity), their intrinsic linker region and size differences might interfere with pharmacokinetics, even though the exact mechanisms behind remain elusive and require more systematic investigation.

A more generalized approach to overcome the pharmacokinetic limitations of the IgBD could be the improvement of the domain itself towards higher affinity to IgG, either by a rational, structure-based design aiming for mutation of affinity-determining amino acid residues or by a random library-based strategy. Such engineering might reasonably be combined with an elimination of T cell epitopes to reduce the potential immunogenicity of the streptococcal IgBD. However, under translational aspects it could be more challenging to assess the PK of Anticalin proteins comprising a “second generation” IgBD, because huIgG/huFcRn double transgenic mouse models are not readily available. On the contrary, the applied hSA/hFcRn double humanized mouse model provides a good basis for the comparable prediction of human PK in case of OX40 Ac-ABD and OX40 Ac-Fc. Here, the expected strong extension of *t*_1/2_ for OX40 Ac-ABD as an effect of longer HSA half-life was accompanied by an even slightly prolonged *t*_1/2_ of OX40 Ac-Fc compared to CD-1 mice, which was surprising in view of the lower affinity of human Fc to huFcRn compared to the pairing human Fc and muFcRn in wild-type mice (Abdiche et al., 2015). Former data from huFcRn transgenic mice show that huIgG or huFc molecules usually expressed shorter plasma half-lives than in wild-type mice. Although we would not exclude a physiological effect due to the additional presence of HSA in this model, the differences between both experiments regarding doses, study length and assay setup could have a contribution as well.

In summary, our study of monomeric immunoglobulin or serum albumin targeting domains for plasma half-life extension of OX40-blocking Anticalin proteins showed that an up to ∼70-fold extension of terminal half-life compared to the naked Anticalin protein is feasible, provided that the respective domain binds its target serum protein with high affinity. The strategy of non-covalent serum protein targeting provides an attractive way for affinity-based plasma half-life tuning of Anticalin proteins in an application-oriented manner.

## Data Availability

The original contributions presented in the study are included in the article/Supplementary Material, further inquiries can be directed to the corresponding author.
