# Supplementary material for: Pharmacokinetic Engineering of OX40-Blocking Anticalin Proteins Using Monomeric Plasma Half-Life Extension Domains
Source: Front Pharmacol. 2021 Oct 25;12:759337. doi: 10.3389/fphar.2021.759337 (PMC8573339; doi:10.3389/fphar.2021.759337)
Supplement: Supplementary file 1 [file DataSheet1.PDF]

Supplemental Figure S1.

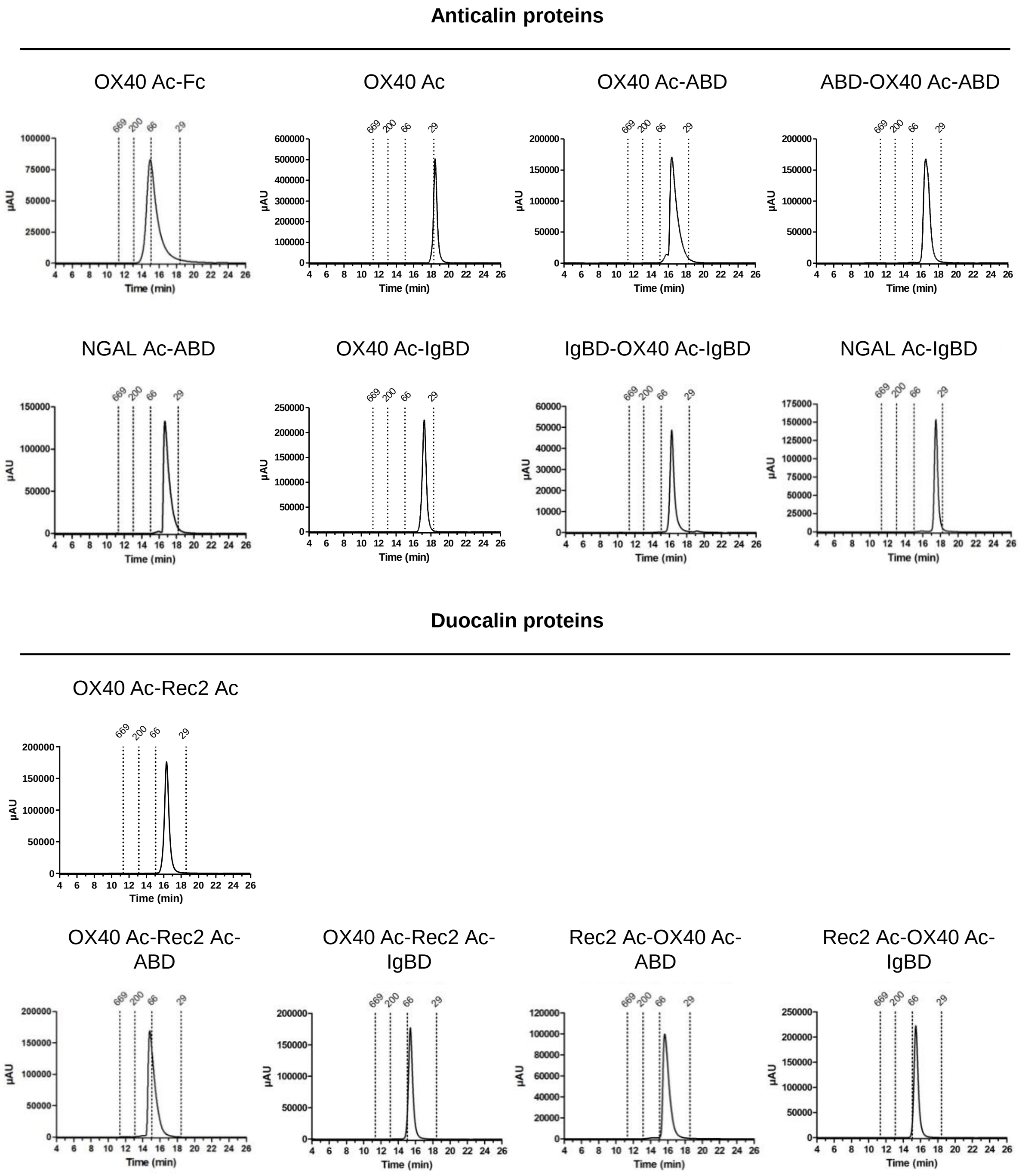

Figure S1. Analytical size exclusion chromatograms (aSEC) of Anticalin proteins. IMAC-purified preparations were separated on a TSKgel® SuperSW mAb HR, 7.8 × 300 mm column (Tosoh) equilibrated in 0.1 M Na_2HPO_4/NaH_2PO_4, 0.1 M Na_2SO_4, pH 6.7. The IMAC eluates of OX40 Ac, OX40 Ac-ABD, ABD-OX40 Ac-ABD, OX40 Ac-IgBD, OX40 Ac-Rec2 Ac, OX40 Ac Rec2 Ac-IgBD, Rec2 Ac-OX40 Ac-ABD and Rec2 Ac-OX40 Ac-IgBD were prior to aSEC additionally purified by preparative gel filtration, although their monomer content after IMAC was already ≥90 %. In all final preparations, a monomer content of ≥97.5 % was proved. The elution times of standard proteins along with their molecular masses in kDa are indicated by dotted lines.

Supplemental Figure S2.

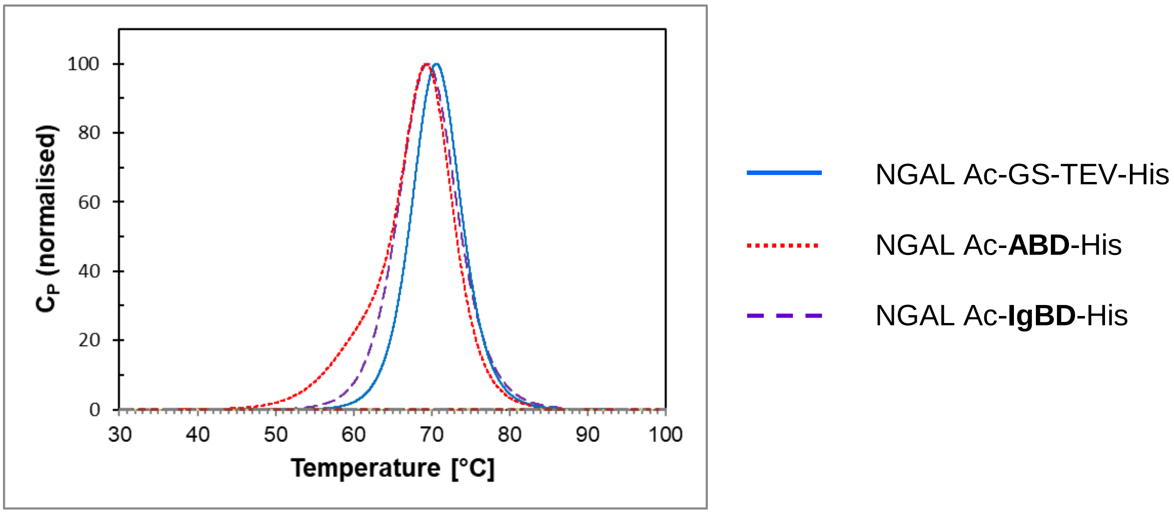

**Molecule**	**T onset (°C)**	**Tm (°C)**
NGAL Ac-GS-TEV-His	57	**70.7**
NGAL Ac-**ABD**-His	47	**62.0; 69.5**
NGAL Ac-**IgBD**-His	54	**69.6**

Figure S2. Differential scanning calorimetry (DSC) of NGAL Anticalin fusion proteins. The thermal unfolding of NGAL Ac-GS-TEV-His, NGAL Ac-ABD-His and NGAL Ac-IgBD-His was analyzed to compare the impact of ABD and IgBD on the stability of Anticalin fusion proteins. While the effects of ABD or IgBD fusion on Tm of NGAL Ac were generally low, the fusion with ABD led to an earlier onset of unfolding in combination with a second unfolding event.

Supplemental Figure S3.

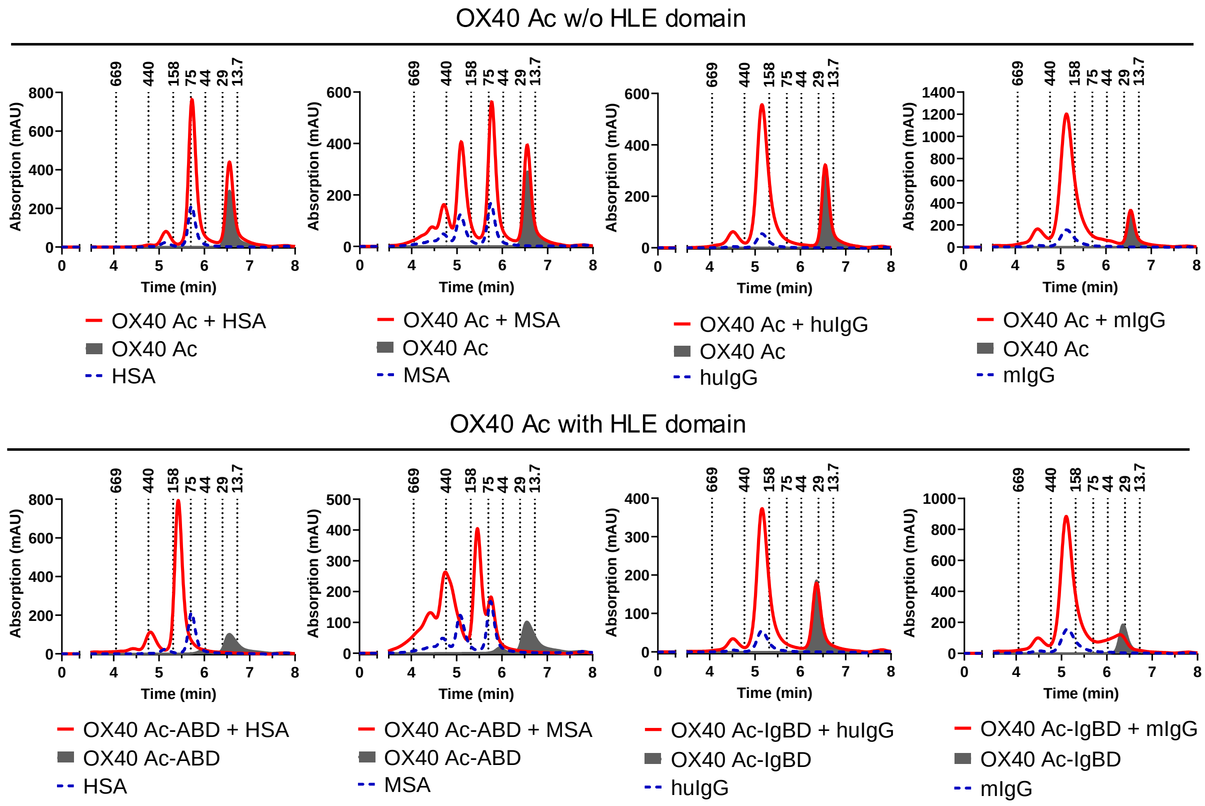

Figure S3. SEC analysis of complex formation with albumin and IgG. The binding of OX40 Ac-ABD to human serum albumin (HSA) or mouse serum albumin (MSA) and OX40 Ac-IgBD to human IgG (huIgG) or mouse IgG (mIgG) was analyzed by SEC (lower graphs). OX40 Ac showed no interaction with the analyzed serum proteins and served as a control (upper graphs). Four µg of each Anticalin protein was incubated with an equimolar amount of the respective serum protein (red chromatograms) or proteins were analyzed separately (dashed and filled chromatograms). The resulting chromatograms are presented as overlays.

Supplemental Table S1.

Table S1. Retention times of Anticalin proteins in heparin chromatography.

Molecule	Retention time (min)
Panitumumab (negative control)	0.70
RNase A (positive control)	16.6
OX40 Ac-Fc	0.93
OX40 Ac	0.94
OX40 Ac-ABD	0.93
ABD-OX40 Ac-ABD	0.93
NGAL Ac-ABD	0.94
OX40 Ac-IgBD	0.94
IgBD-OX40 Ac-IgBD	n. d.
NGAL Ac-IgBD	0.94
OX40 Ac-Rec2 Ac	0.92
OX40 Ac-Rec2 Ac-ABD	0.92
OX40 Ac-Rec2 Ac-IgBD	0.92
Rec2 Ac-OX40 Ac-ABD	0.92
Rec2 Ac-OX40 Ac-IgBD	0.83

Supplemental Table S2.

Table S2. EC_{50} values of Anticalin proteins binding to OX40-overexpressing CHO cells in absence or presence of 2 % human serum (normalized datasets from four experiments, mean and 95 % C.I.).

Molecule	EC_{50} ($\times 10^{-9}$ M)	95 % C.I. ($\times 10^{-9}$ M)	+ HuSe; EC_{50} ($\times 10^{-9}$ M)	+ HuSe; 95 % C.I. ($\times 10^{-9}$ M)
OX40 Ac	2.9	1.7 to 5.1	13.5	8.2 to 21.8
OX40 Ac-ABD	26.9	19.8 to 36.4	1.3	0.5 to 3.4
ABD-OX40 Ac-ABD	12.6	7.0 to 22.1	1.5	0.5 to 4.2
NGAL Ac-ABD	n. b.	n. b.	n. b.	n. b.
OX40 Ac-IgBD	6.8	4.2 to 11.1	20.6	3.3 to 100
IgBD-OX40 Ac-IgBD	3.0	1.5 to 5.8	9.4	1.8 to 62
NGAL Ac-IgBD	n. b.	n. b.	n. b.	n. b.
OX40 Ac-Rec2 Ac	9.0	4.3 to 19.2	11.7	6.1 to 22.2
OX40 Ac-Rec2 Ac-ABD	13.6	6.2 to 28.8	1.5	0.6 to 3.6
OX40 Ac-Rec2 Ac-IgBD	5.1	2.7 to 9.5	21.0	4.2 to 99
Rec2 Ac-OX40 Ac-ABD	10.1	5.2 to 19.4	2.9	1.4 to 5.6
Rec2 Ac-OX40 Ac-IgBD	5.1	2.5 to 10.6	65	16.5 to 206

HuSe human serum

n. b. no binding detected

Supplemental Table S3.

Table S3. IC_{50} values (nM) of inhibition of luciferase activity measured from NFκB-Luc2/OX40 Jurkat reporter cells in consequence of OX40 receptor blocking by Anticalin and Duocalin proteins (one representative experiment out of three independent experiments, mean and 95 % C.I.).

Molecule	IC_{50} ($\times 10^{-9}$ M)	95 % C.I. ($\times 10^{-9}$ M)
OX40 Ac	0.96	0.69 to 1.33
OX40 Ac-ABD	0.49	0.36 to 0.65
ABD-OX40 Ac-ABD	0.46	0.34 to 0.61
NGAL Ac-ABD	n. i.	n. i.
OX40 Ac-IgBD	0.89	0.63 to 1.24
IgBD-OX40 Ac-IgBD	1.04	0.76 to 1.42
NGAL Ac-IgBD	n. i.	n. i.
OX40 Ac-Rec2 Ac	0.81	0.40 to 1.41
OX40 Ac-Rec2 Ac-ABD	1.01	0.77 to 1.31
OX40 Ac-Rec2 Ac-IgBD	1.18	0.91 to 1.50
Rec2 Ac-OX40 Ac-ABD	1.20	0.88 to 1.61
Rec2 Ac-OX40 Ac-IgBD	1.83	1.21 to 2.79

n. i. no inhibition

Supplemental Table S4.

Table S4. IC_{50} values (nM) of inhibition of IL-2 release by panT cells from four different donors in consequence of OX40 receptor blocking by Anticalin and Duocalin proteins in a co-culture setting.*

Molecule	Donor A IC_{50} ($\times 10^{-9}$ M)	Donor B IC_{50} ($\times 10^{-9}$ M)	Donor C IC_{50} ($\times 10^{-9}$ M)	Donor D IC_{50} ($\times 10^{-9}$ M)
OX40 Ac	9.1	11.1	15.4	5.7
OX40 Ac-Rec2 Ac	2.7	6.1	10.2	-
OX40 Ac-ABD	3.2	2.5	5.6	2.8
OX40 Ac-Rec2 Ac-ABD	-	-	10.7	-
NGAL Ac-ABD	n. i.	n. i.	n. i.	n. i.

- insufficient sigmoidal curve shape for IC_{50} calculation

n. i. no inhibition

* panT cells from donor A, B, C or D were cultivated on anti-CD3 antibody-coated plates at a ratio 3:1 with mitomycin C treated Flp-In-CHO::vector cells and T cell alloreactivity was stimulated by addition of 4 nM OX40 ligand in presence of anti-CD-28 antibody; titrated Anticalin proteins were tested for inhibition of IL-2 release
